# Post-operative atrial fibrillation is associated with a pre-existing structural and electrical substrate in human right atrial myocardium

**DOI:** 10.1016/j.ijcard.2016.06.249

**Published:** 2016-10-01

**Authors:** Junaid A.B. Zaman, Leanne Harling, Hutan Ashrafian, Ara Darzi, Nigel Gooderham, Thanos Athanasiou, Nicholas S. Peters

**Affiliations:** a Myocardial Function, National Heart & Lung Institute, Hammersmith Hospital, Imperial College London, UK; b Department of Surgery and Cancer, St Mary's Hospital, Imperial College London, UK; c Department of Biomolecular Medicine, South Kensington Campus, Imperial College London, UK; d Cardiovascular Medicine, Stanford University, Palo Alto, USA

**Keywords:** Atrial fibrillation, Post-operative, Calcium handling, Electrograms, Dominant frequency

## Abstract

**Background:**

Post-operative atrial fibrillation (POAF) is a major health economic burden. However, the precise mechanisms in POAF remain unclear. In other forms of AF, sites of high dominant frequency (DF) in sinus rhythm (SR) may harbour ‘AF nests’. We studied AF inducibility in relation to substrate changes using epicardial electrograms and cardiomyocyte calcium handling in the atria of AF naïve patients.

**Method:**

Bipolar electrograms were recorded from the lateral right atrial (RA) wall in 34 patients undergoing coronary surgery using a high-density array in sinus rhythm (NSR). RA burst pacing at 200/500/1000 ms cycle lengths (CL) was performed, recording episodes of AF > 30 s. Co-localised RA tissue was snap frozen for RNA and protein extraction.

**Results:**

Electrograms prolonged during AF (76.64 ± 29.35 ms) vs. NSR/pacing (p < 0.001). Compared to NSR, electrogram amplitude was reduced during AF and during pacing at 200 ms CL (p < 0.001). Electrogram DF was significantly lower in AF (75.87 ± 23.63 Hz) vs. NSR (89.33 ± 25.99 Hz) (p < 0.05), and NSR DF higher in AF inducible patients at the site of AF initiation (p < 0.05). Structurally, POAF atrial myocardium demonstrated reduced sarcolipin gene (p = 0.0080) and protein (p = 0.0242) expression vs. NSR. Phospholamban gene and protein expression was unchanged. SERCA2a protein expression remained unchanged, but MYH6 (p = 0.0297) and SERCA2A (p = 0.0343) gene expression was reduced in POAF.

**Conclusions:**

Human atrial electrograms prolong and reduce in amplitude in induced peri-operative AF vs. NSR or pacing. In those sustaining AF, high DF sites in NSR may indicate ‘AF nests’. This electrical remodelling is accompanied by structural remodelling with altered expression of cardiomyocyte calcium handling detectable before POAF. These novel upstream substrate changes offer a novel mechanism and manifestation of human POAF.

## 1 Background

*De novo* post-operative atrial fibrillation (POAF) affects approximately 30–60% of patients undergoing cardiac surgery [1], [2]. It is associated with increased post-operative morbidity, mortality, and significantly worse long-term outcomes [3], [4], [5], [6]. The make-up or existence of any predisposing atrial substrate for POAF remains poorly understood.

Methods to detect upstream substrate changes have proved challenging, but it is likely these changes exist in even ‘lone AF” [7]. One electrical substrate feature is putative “AF nests” [8] whereby “fibrillar’ myocardium is more likely to harbour sites of AF maintenance than so called “compact” myocardium in patients with persistent AF. These sites characteristically display a broad power spectral density (PSD) of the sinus rhythm electrogram when analysed using Fast Fourier Transform (FFT) and occur in both atria. The applicability of these findings in earlier forms of AF such as POAF is unknown.

This study addresses the hypothesis that acute onset POAF is associated with antecedent changes in the make-up of the atrial epicardial electrograms and calcium handling proteins that give rise to a vulnerable substrate in which peri-operative triggers result in clinically significant AF. This is motivated by several lines of reasoning. Firstly, work from our group has discovered that changes in intra-operative paced electrogram changes are able to identify a population who subsequently go on to develop post-operative AF, related to changes in connexin expression [9]. Secondly, we also reported changes apparent in sinus rhythm electrograms in an “AF naïve” patient group as potential indicators of a predisposed substrate which the operative ‘trigger’ will interact with to produce POAF [10]. However, there is a lack of upstream atrial structure/function studies to detect, prevent or treat POAF especially in subjects with no antecedent AF history.

Third, in addition to these changes in connexin expression and quantity, there is growing evidence to suggest that structural and electrical remodelling occur at a transcriptional level when considering the mechanism behind abnormal atrial cardiomyocyte electrophysiology. In particular, alterations to intracellular calcium handling proteins may act as a key component of POAF pathogenesis, as with other forms of AF [11]. Reduced SERCA2A and/or increased ryanodine receptor (RYR3) gene expression may directly increase cytosolic Ca^2 +^ through increased reuptake [12], [13], [14] and sarcoplasmic reticulum (SarcR) Ca^2 +^ release respectively [15]. Conversely, compensatory mechanisms to increase SarcR Ca^2 +^ re-uptake and restore Ca^2 +^ homeostasis have also been associated with AF, including downregulation of sarcolipin (SLN), which itself negatively regulates SERCA2A [16]. Indirectly, dysregulation of RhoC signalling may promote AF pathogenesis through both perturbation of ROCK signalling pathways, and through MAPK and PI3K signalling which in turn may alter SarcR Ca^2 +^ uptake [17]. Similarly, increased expression of the pro-oxidant monoamine oxidase B may produce intracellular Ca^2 +^ overload through generation of H_2_O_2_, mitochondrial damage and increased mitochondrial Ca^2 +^ release [18].

We set out to study this hypothesis in a group of patients with no previous AF history, forming one of the furthest upstream atrial structure function studies carried out in the literature to date.

## 2 Methods

### 2.1 Patient selection and recruitment

This study complies with the Declaration of Helsinki, and the study was approved by the local and regional research ethics committee (Ref: 09/H0711/23). Informed consent was obtained from all subjects. Between November 2010 and September 2011, thirty-four patients undergoing non-emergent, on-pump coronary artery bypass grafting (CABG) at Imperial College Healthcare NHS Trust, were prospectively selected to participate in this study. Emergency cases, those requiring adjunctive procedures (e.g. valve repair or replacement), patients with a prior history of any cardiac arrhythmia, thyroid disease, those taking anti-arrhythmic agents, or undergoing surgery with mini-cardiopulmonary bypass (CPB) systems were excluded.

All patients underwent continuous Holter (Novocor Vista 5 lead system, 2 channel recording) monitoring from the time of admission to the time of surgery (12–24 h). Atrial fibrillation was defined according to Heart Rhythm Society Guidelines [19]. Post-operative atrial fibrillation (POAF) was defined as new onset AF following CABG surgery in patients with pre-operative Holter recordings demonstrating sinus rhythm and no prior history of the arrhythmia. All episodes of AF, atrial flutter, or tachycardia of at least 30 s duration were documented. Only AF episodes > 30 s were categorised as AF positive (group 2) [19]. Patients were grouped retrospectively (following Holter analysis after discharge) according to the absence (Group 1) or presence (Group 2) of POAF.

### 2.2 Electrophysiological study

The intra-operative electrophysiological protocol was designed to minimise delay in surgery or deleterious effects to the patient should AF be induced. Immediately prior to establishing cardio-pulmonary bypass, a high-density Inquiry AFocus II catheter (Irvine Biomedical, St Jude Medical, Minnesota, USA) was placed by the surgeon on the lateral right atrial wall. Stability of contact was ensured by consistent paced capture with high signal to noise ratio on bipolar electrograms. Pacing was always performed at twice the pacing threshold.

Baseline bipolar atrial electrograms were recorded during sinus rhythm and pacing at 500 ms cycle length pacing from an outer pair of electrodes. A steady-state recording of at least 10 consecutive beats was obtained. Electrograms were digitised and stored using BARD (Boston Scientific, USA) at standard sampling rates of 1 kHz and bandpass filtered between 30.0 Hz and 300 Hz.

Atrial fibrillation (AF) was then induced by burst pacing from the same lateral electrode pair to ensure consistent pacing direction and planar wavefronts. Pacing was performed at 200 ms cycle length for 5 s (four bursts) and then 10 s (two bursts) until AF occurs. AF was considered significant if sustained for greater than 30 s [20], [21].

### 2.3 Electrogram analysis

Intra-operative bipolar electrograms were analysed for the following parameters in an automated software package written in Labview based on previous studies [8], [22], [23]: duration, peak-to-peak amplitude, dominant frequency (DF) and activation time. Electrogram duration was calculated as time from first deviation from baseline to return [24]. Activation time was defined as the time from first to last activation of the AFocus. Conduction velocity (CV) was calculated from the manually annotated activation times using Matlab [25]. This enabled visualisation of propagation across the AFocus II as an isochronal map and estimated CV both using planar and circular conditions. An overall direction of propagation was also calculated and maps visually assessed for atrial conduction patterns. We present mean electrophysiological data from at least 6 consecutive beats. All electrophysiological analyses were conducted blinded to the patient POAF status.

### 2.4 Laboratory methods

#### 2.4.1 Tissue sampling

Atrial tissue biopsies were taken (without the use of electrocautery) from the free wall of the right atrium prior to cannulation and institution of cardiopulmonary bypass. This corresponded to the site of the electrogram recordings at a consistent site at the lateral border of the AFocus catheter. All biopsies were taken prior to pacing protocols to avoid confounding effects of AF induction on calcium handling proteins. Due to ethical considerations of taking left atrial biopsies in patients undergoing routine coronary surgery, with no need for left atrial access, only right atrial studies were performed.

#### 2.4.2 RNA extraction

Whole RNA was extracted from atrial tissue using TRIzol® Reagent as described previously [26]. RNA quality and concentrations were assessed using the Nanodrop 1000 spectrophotometer and Agilent 2100 Bioanalyser. All RNA integrity numbers (RIN) were > 7.5.

#### 2.4.3 RT-qPCR

mRNA levels were quantified using specific TaqMan™ qPCR gene expression assays for the following gene targets: SLN, PLN, Triadin (TRDN), SERCA2A, MYH6 (Applied Biosystems). For each study probe an identical PCR reaction was also carried out using ribosomal U6 as a control gene.

#### 2.4.4 Reverse transcription

Reverse transcription was performed utilising a high-capacity cDNA universal RT kit in accordance with the manufacturer protocol (TaqMan, Applied Biosystems, Life Technologies, Paisley, UK). A total of 10 μl RT master mix was added to 5 μl of sample containing 1 μg total RNA. On completion of the RT reaction, resultant cDNA was either used immediately or stored at − 80 °C.

#### 2.4.5 qPCR reaction

qPCR reactions were performed using TaqMan gene expression assays according to manufacturer protocol. A total reaction volume of 10 μl was used for qPCR and an identical control PCR reaction (U6) carried out for each sample reaction. All reactions were carried out in three technical replicates using the Applied Biosystems 7500 fast Real-Time PCR System [27]. Amplification plots were examined for adequate amplification and a delta R_n_ threshold value of 0.2 (within the exponential phase of amplification) was set to ensure comparability across plates. C_t_ values were recorded for both samples and controls and compared using the ΔΔC_t_ method.

#### 2.4.6 Western blotting

Protein was extracted from RA tissue samples (25–50 mg) using RIPA buffer with added protease inhibitor cocktail (*Sigma*). Prior to commencing western blotting, protein concentrations were determined by BCA assay. Proteins were separated by SDS–polyacrylamide gel electrophoresis (10-well 10% NuPAGE® Bis-Tris Precast Gels (Life Technologies) with MES running buffer) and blotted onto a 0.45 μm Protran® membrane. Successful protein transfer was confirmed with Ponceau S stain. Membranes were blocked for 1 h at room temperature in 5% milk blocking buffer and then incubated with primary antibody overnight at 4 °C (Rabbit polyclonal Anti-Sarcolipin (#ABT13, Millipore UK), Mouse monoclonal Anti-Phospholamban 2D12 (#ab2865, Abcam), and Mouse monoclonal Anti-SERCA2A ATPase (2A7-A1; #ab2861, Abcam). Membranes were then washed in PBS-T (PBS with 0.01% Tween) and incubated for 1 h in blocking buffer containing secondary antibody. After washing, protein bands were visualised using enhanced chemiluminescence (Thermo Scientific, Rockford, IL). Quantitation of protein concentrations was preformed using Kodak Image Station 2000M analysis software. Normalisation of protein loading was performed against β-Actin (primary mouse β-Actin (Abcam)).

### 2.5 Statistical analysis

Inter-group comparisons were performed using student t-test if two groups, or one-way ANOVA if multiple groups. Statistical significance is reported if p < 0.05. All calculations were performed using Prism 6.0 (GraphPad, La Jolla, USA).

## 3 Results

34 patients undergoing non-emergent, on-pump CABG were recruited. 21 did not develop POAF and were classified into Group 1, 13 patients developed POAF and were classified into Group 2. The mean time to onset of AF was 2.5 days.

### 3.1 Pre-operative demographics

A summary of the pre-operative demographics is shown in Table 1. Echocardiographic parameters were also available for the majority of patients and are shown in Table 2. No differences were observed in age, gender, pre-operative risk stratification score (logEuroSCORE), echocardiographic parameters or other recognised cardiovascular risk factors between the two groups. Anti-arrhythmic therapy, especially beta-blockers which may reduce POAF, was similar between groups.

### 3.2 Epicardial electrograms prolong and reduce in amplitude prior to onset of AF

**Fig. 1:**
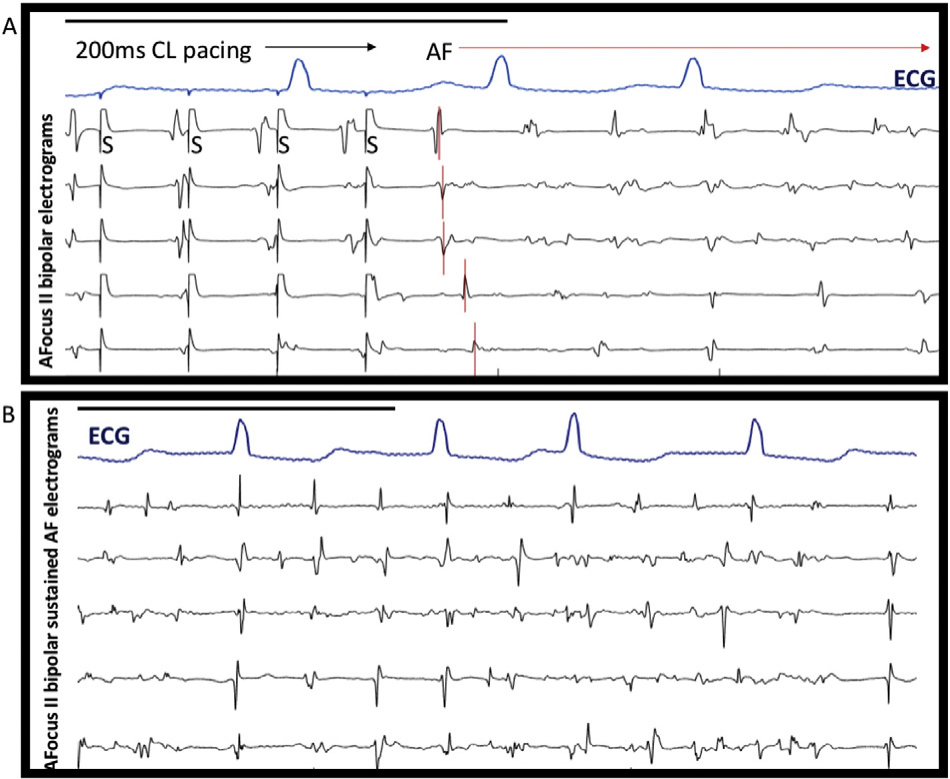
Induced AF shows rapidly changing complex electrogram morphology in 72-year-old man with no history of AF. A) Episode of induced intra-operative AF immediately after atrial pacing at 200 ms CL from bipole 5/6. First beat of induced AF annotated in red, showing sequential propagation across catheter bipoles. S demarcates pacing stimulation artefact. B) Select bipoles after 30 s of sustained AF show marked electrogram complexity. Black bars indicate 1000 ms in both panels.

**Fig. 2:**
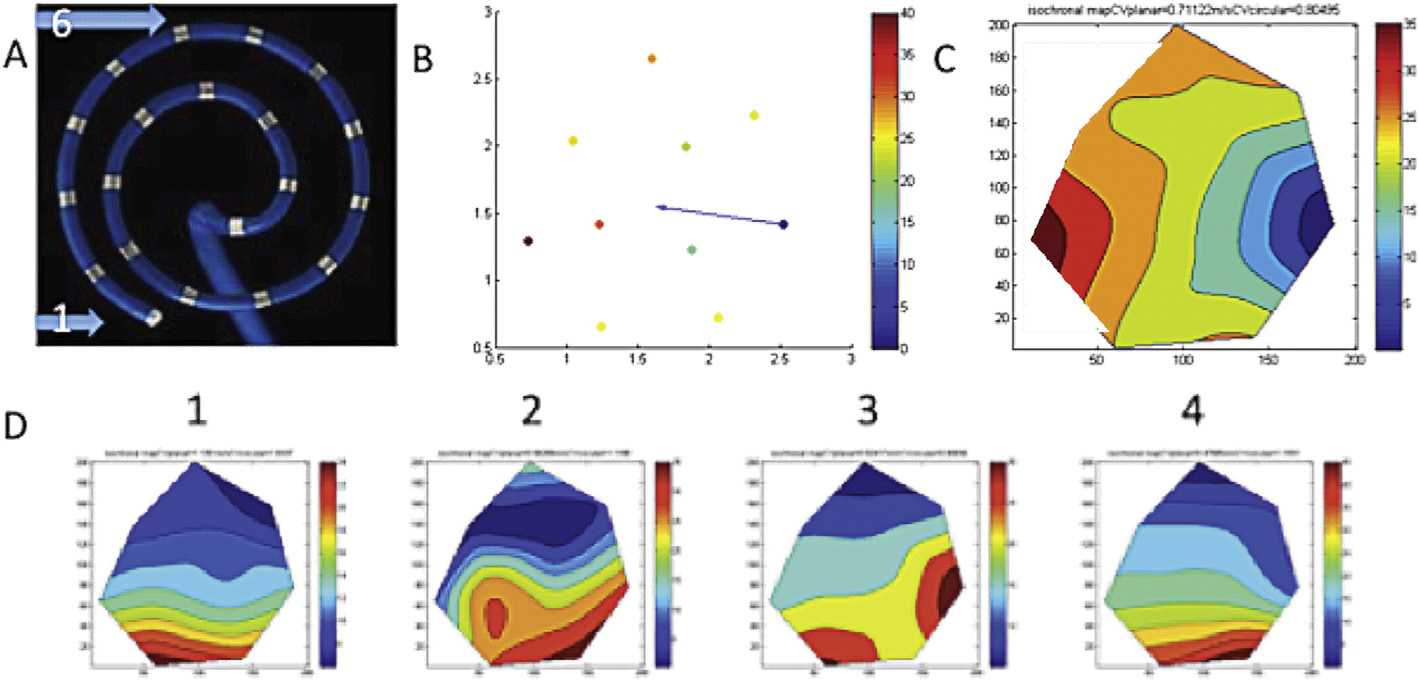
Activation maps of acutely induced human AF show coherent wave propagation. A) A Focus II with labelled electrodes 1 and 6. B) Manually assigned activation times on spiral Matlab schematic with colour coding and overall activation vector labelled (blue arrow). C) Isochronal map from activation times showing smooth propagation across AFocus area with calculated CV at top. D) 4 cycles of AF showing relatively consistent activation patterns using above steps.

Atrial electrograms of induced AF showed variable morphology with highly fractionated components (Fig. 1). Electrogram duration was significantly prolonged in AF (76.64 ± 29.35 ms) compared to pacing or normal sinus rhythm (NSR) (52.89 ± 13.87 ms, p < 0.001). There was a progressive decrease in amplitude of electrograms from NSR (1.38 ± 0.70 mV) through pacing (500 ms 1.11 ± 0.57 mV, 200 ms 0.91 ± 0.61 mV) until AF, which had the lowest amplitude (0.85 ± 0.51 mV, p < 0.001 vs. NSR). This was associated with a prolonged activation time during rapid pacing (200 ms CL 29.41 ± 16.23 ms vs. 1000 ms CL 22.64 ± 8.40 ms, p < 0.05)) suggestive of slowed conduction [22]. However when the mean wavefront propagation velocity was calculated there was no significant difference between these groups, but a trend towards slowed CV at rapid pacing (200 ms) versus normal sinus rhythm (78.5 ± 37.8 cm/s vs. 92.1 ± 20.6 cm/s, p = 0.07). Our method of calculating CV showed good correlation between planar vs. circular wavefront conditions. Activation maps showed mostly smoothly propagating simple wavefronts, consistent with early AF with minimal remodelling (Fig. 2) [28].

### 3.3 Electrogram dominant frequency in sinus rhythm is higher than AF

**Fig. 3:**
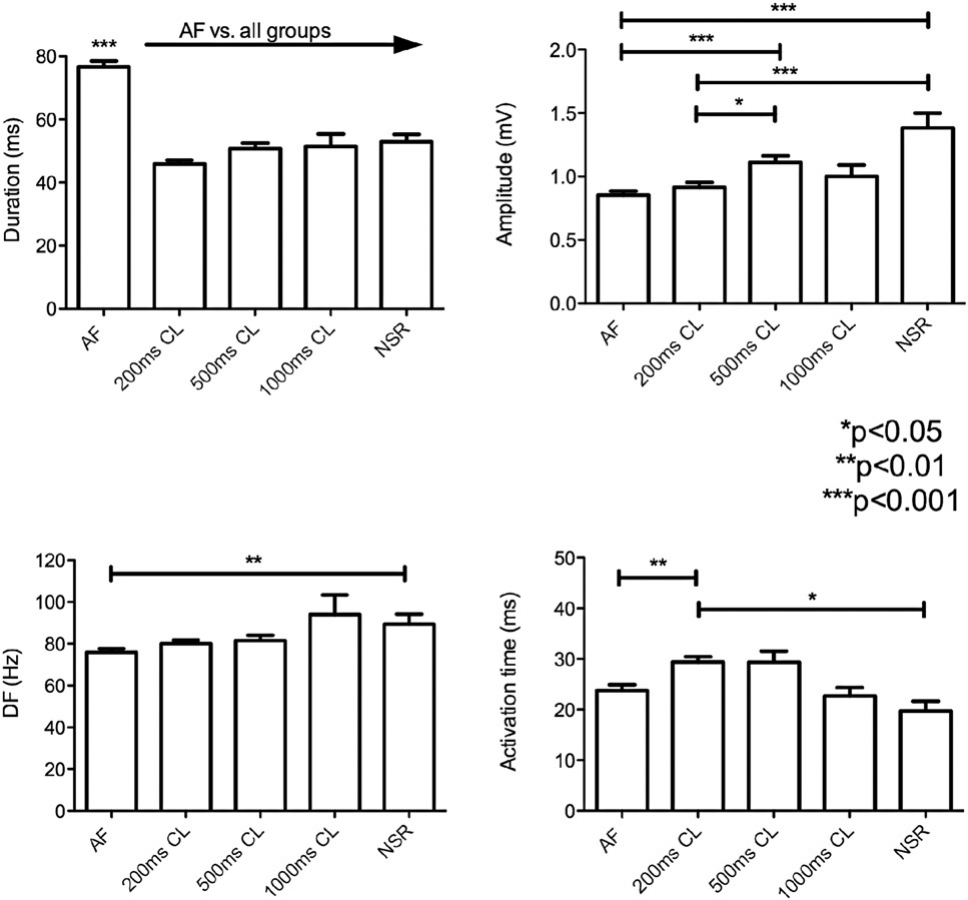
Summary of intra-operative electrogram findings when analysed by pacing rate or normal sinus rhythm (NSR). For further details please refer to text.

**Fig. 4:**
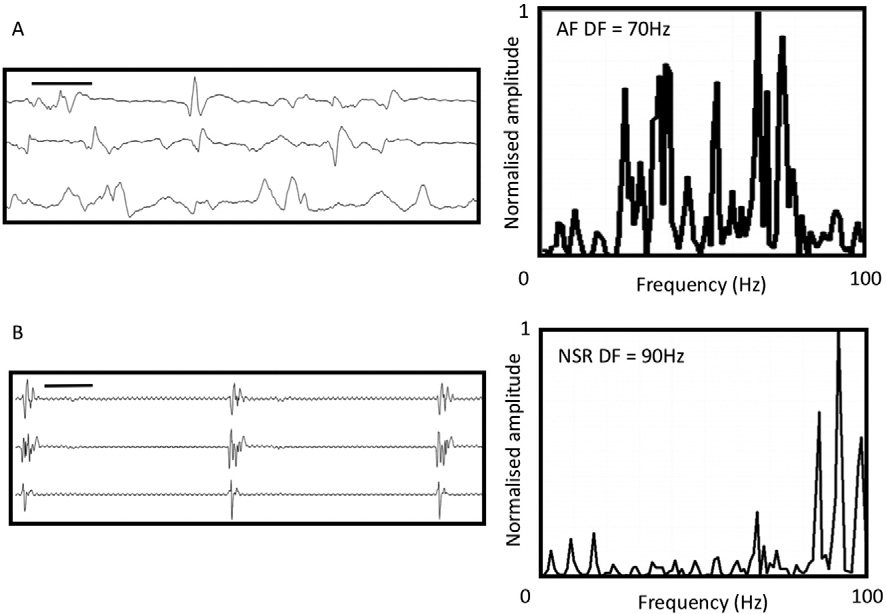
Examples of AF and sinus electrograms from a 73-year-old man with induced intra-operative AF. A) 3 bipoles from AFocus II after sustained AF for 30 s with corresponding Fast Fourier Transform (FFT) spectral analysis show dominant frequency peak at 70 Hz. B) Same bipoles during sinus rhythm in the same patient prior to AF induction. High frequency fractionation is seen in top two channels. Overall FFT showed a frequency peak at 90 Hz. Black bar represents 1000 ms in both electrogram panels. Mean data is shown in Fig. 3.

**Fig. 5:**
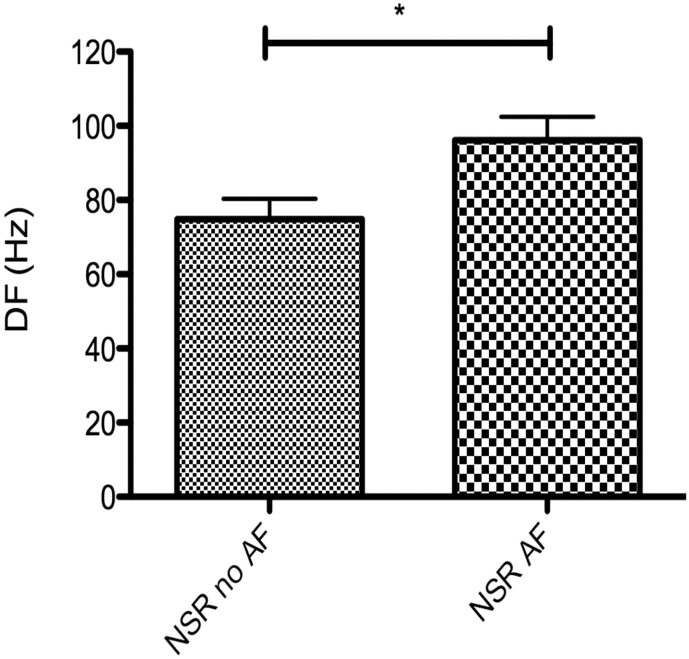
Dominant frequency (DF) of normal sinus rhythm (NSR) electrograms at the site of AF initiations is higher in patients who sustain AF (NSR AF) intra-operatively (*p < 0.05).

Fourier analysis of the electrogram showed a higher mean dominant frequency in sinus rhythm than in AF (89.3 ± 4.9 Hz vs. 75.9 ± 1.8 Hz respectively, p = 0.006). The pacing rate affected the magnitude of this result, with the largest spectral differences occurring between AF vs. NSR and 1000 ms pacing. A summary of all electrogram findings is shown in Fig. 3. An example of AF and sinus rhythm electrograms with corresponding power spectral density plots of the electrogram is shown in Fig. 4. Patients able to be paced intra-operatively into AF had higher DF at the site of AF initiations during sinus rhythm than those who did not (96.2 ± 6.2 Hz vs. 74.9 ± 5.6 Hz, p = 0.04) (Fig. 5).

### 3.4 Gene and protein expression of intracellular calcium handling proteins

**Fig. 6:**
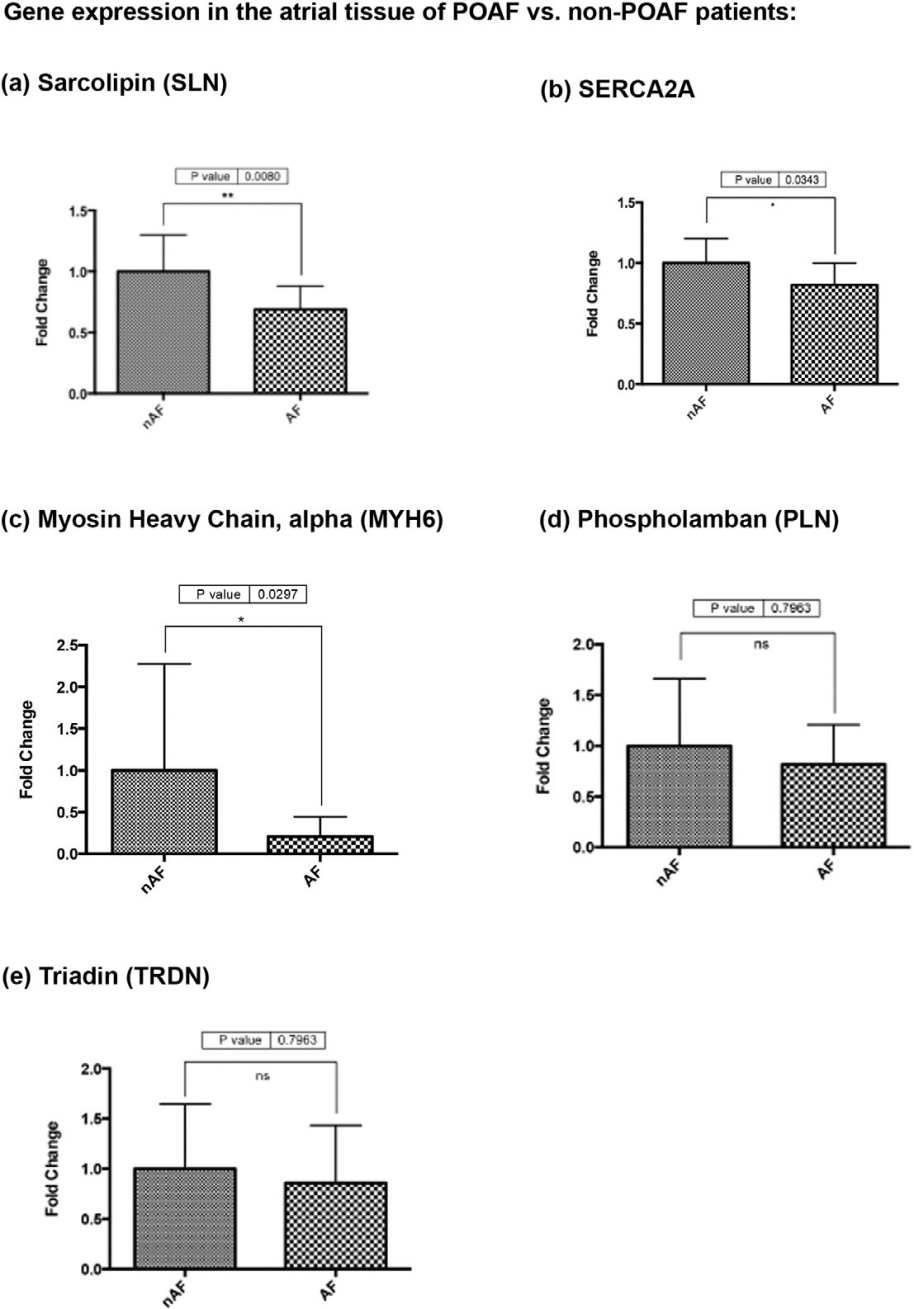
RT-qPCR results showing significantly lower (a) sarcolipin (SLN) (p = 0.0080); (b) SERCA2A (p = 0.0343), and (c) MYH6 (p = 0.0297) gene expression in the right atrial tissue of POAF compared to non-POAF. No significant difference was observed in (d) phospholamban (PLN) (p = 0.7963), or (e) triadin (TRDN) (p = 0.7963) gene expression.

POAF was found to be associated with a significant reduction in the expression of SLN mRNA (p = 0.0080) as well as a less significant reduction in MYH6 (p = 0.0297) and SERCA2A (p = 0.0343) gene expression. No significant difference was observed in PLN (p = 0.7963) or Triadin (p = 0.7963) gene expression (Fig. 6).

**Fig. 7:**
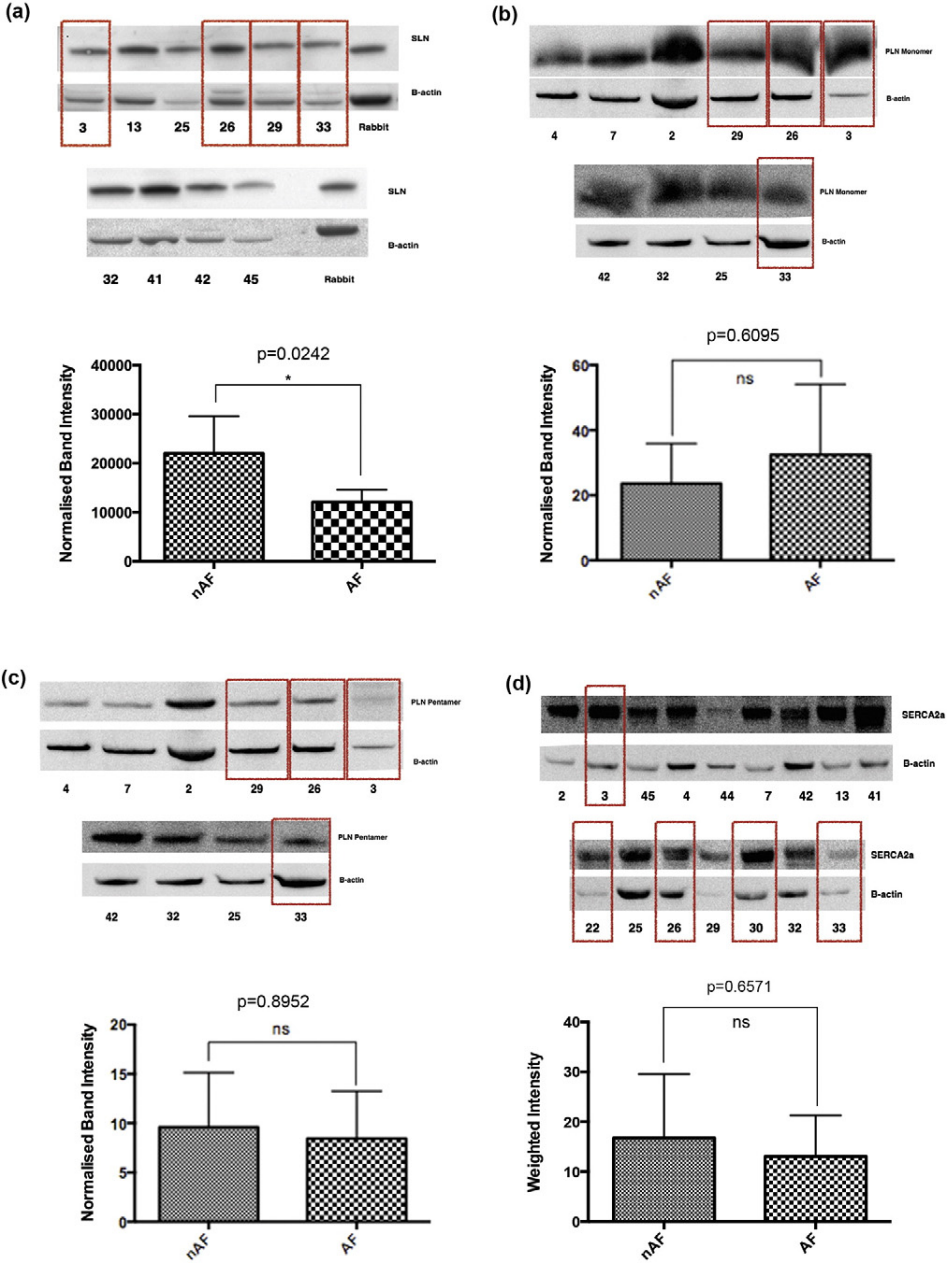
Western blot demonstrating: (a) significantly lower sarcolipin (SLN) (p = 0.0242), and unchanged (b) monomeric phospholamban (p = 0.6095), (c) pentameric phospholamban (p = 0.8952), and (d) SERCA2A (p = 0.6751) protein expression in the right atrial tissue of patients developing post-operative atrial fibrillation (POAF) compared to those maintaining sinus rhythm. Boxes denote samples from POAF patients.

Sarcolipin (SLN) protein levels were evaluated in the atrial tissue of a subgroup of 4 POAF and 7 non-POAF patients. Patients developing POAF had a significantly lower level of SLN protein expression in atrial tissue samples taken at the time of surgery when compared to non-POAF patients (p = 0.0242) (Fig. 7a).

No significant difference was seen in the expression of the regulator protein phospholamban (PLN) (monomer p = 0.6095; pentamer p = 0.8952) or in the overall expression of SERCA2a (p = 0.6571) (Fig. 7b–d). Insufficient atrial tissue sample was available to allow for evaluation of atrial myosin heavy chain 6 (cardiac muscle, alpha isoform) protein expression in the current study.

## 4 Discussion

This study is the first to identify that intraoperative electrograms obtained during sinus rhythm in patients with no prior history of AF have a higher dominant frequency (DF) than those obtained during AF. Furthermore, patients in whom AF was inducible demonstrated higher SR DF electrograms than those in whom AF was non-inducible. These electrical changes were coupled with altered cardiomyocyte calcium handling apparent in POAF atria prior to the onset of arrhythmia, as evidenced by changes in SLN, MYH6 and SERCA2a gene expression and reduced sarcolipin protein expression.

### 4.1 AF ‘nests’ in POAF patients?

Despite no prior history of any cardiac arrhythmia in our patients, there was a significant increase in the DF of the sinus rhythm electrogram when compared to the electrogram in AF. Whilst this is compatible with the ‘AF nest’ theory, whereby sources in the atrium harbouring AF are detectable at baseline in patients undergoing AF ablation, these findings are the first in such an upstream, unremodelled cohort [8]. In that study, Pachon et al. found a detectable AF nest in only 1 of the 6 controls (with no prior history of AF), with AF only inducible in this patient. This has parallels to the findings we present here, whereby patients inducible into AF had higher electrogram DF in sinus rhythm than those who were not.

Variations in connexin expression and complexity of wavefront propagation have previously been shown to indicate those at risk of post-operative AF [9], [10]. However, these changes were mainly observed in during paced rhythms. Our findings are the furthest ‘upstream’ electrogram changes detected so far in human AF. We have shown that patients with no clinical history of AF possess the substrate to maintain AF, and that this may be identifiable by high DF on spectral analysis of intra-operative electrograms.

Our site of recording in the lateral RA was consistent with previous work showing that lateral RA AF nests can occur [8], [29]. This would avoid potential interactions with ganglionated plexi as there are no discrete ganglia in that area [30]. The mechanisms for the AF nest in this site deserve further prospective study, but may involve the crista terminalis or the presence of thick-thin fibre transitions, which may act as AF anchors in the left atrium [31]. Notably in the original description of AF nests, 47% of patients had these at the lateral RA wall near the crista terminalis. Furthermore, differences between electrogram morphology from endo-epicardial recordings have been shown in patients with no history of prior AF [32].

### 4.2 Conduction time prolongs with rapid pacing prior to AF initiation

Rapid pacing to induce AF causes prolongation of conduction time, which correlates with conduction slowing [22]. Although both planar and circular wavefront conditions assumed may be overly simplistic, there was good agreement between both methods. Furthermore, similar assumptions have limited previous in vivo atrial CV measurements, especially during AF. By showing both conditions give similar results, we demonstrate the validity of the CV calculations and further confirm that the episodes of acutely induced peri-operative AF were not due to intrinsic pre-existing conduction defects. In this study we nonetheless observed prolonged activation time with coherent propagation, demonstrated by broad smooth wavefronts on activation maps.

When considering the potential underlying cellular mechanisms behind electrogram fractionation and high dominant frequencies in POAF patients, potential structural mechanisms for AF nests include reduced connexin expression, underlying autonomic ganglia [30], localised microfibrosis [33] and functional causes of fractionation [34]. Functional causes would not be present during sinus rhythm, but are important in AF initiation as conduction slows. We have recently demonstrated this cause of electrogram fractionation in a goat model of tachy-paced AF secondary to altered gap junction remodelling [35]. Significantly, in addition to the above documented electrical substrate factors, we found perturbed calcium handling in POAF pathogenesis.

### 4.3 Electrical remodelling: dysregulation of cardiomyocyte calcium handling in POAF atria

It is known that intracellular calcium overload can lead to action potential alternans and summated extracellular potentials (or after-depolarisations) [36], which may lead to altered electrogram composition. Furthermore, rapid pacing can itself lead to cellular calcium overload, thereby forming an important arrhythmic focus. The atria of POAF patients demonstrated a significant reduction in sarcolipin (SLN) gene and protein expression when compared to the atria of patients maintaining sinus rhythm. Sarcolipin is a small 4 kDa, 31 amino acid protein (encoded by the SLN gene, location 11q22), expressed predominantly in atria, that acts to inhibit the sarcoplasmic reticulum ATPase (SERCA) independent to its primary regulatory protein phospholamban (PLN). This correlates with findings in patients with chronic AF as evidenced by Shanmugam et al. who observed a similar significant decrease in sarcolipin protein expression [16], [37]. Furthermore, our results also corroborate previous findings that this mechanism of SERCA regulation in AF occurs independent to the activity of the regulatory protein phospholamban, the expression of which remains unchanged [16].

Through inhibition of SERCA activity, SLN acts to reduce cellular reuptake of Ca^2 +^ into the sarcoplasmic reticulum (SarcR). Knockout of SLN removes this inhibition of SERCA2a in cardiac muscle, enhances SarcR Ca^2 +^ reuptake and promotes SarcR Ca^2 +^ overload [36], [38]. This in turn shortens the effective refractory period and thus may increase susceptibility to AF. Furthermore, increased SarcR calcium load may also lead to diastolic calcium leak giving rise to the potential for delayed after depolarisations and thus triggered AF [39]. Beyond this, SLN knockout mice also display increased cardiomyocyte L-type Ca2 + channel activity hence greater Ca2 + influx in response to an action potential.

The observed reduction in SLN expression in our POAF patient cohort may therefore represent an adaptive mechanism to counter increased intracellular calcium and preserve SarcR calcium stores through removal of inhibition of SERCA2a. Furthermore, it is possible that the accompanying reduction in MYH6 gene expression represents an MHC isoform switch to reduce α-MHC and increase β-MHC representing a hallmark of cellular reversion to a foetal phenotype [40]. Indeed, β-MHC isoforms are less sensitive to intracellular calcium, and require an approximately 50% higher intracellular free calcium concentration to achieve half-maximal tension than α-MHC [41]. Therefore an MHC class switch may dampen the contractile response to intracellular calcium overload and ultimately prevent contractile failure in response to chronic calcium overload and metabolic stress [40].

Together these changes suggest that POAF may be associated with a basal state of cellular calcium overload prior to the onset of the arrhythmia. Although the exact mechanism for this remains unclear, a number of factors previously associated with AF pathogenesis may be responsible for this increase in intracellular Ca^2 +^ including increased oxidative stress, mitochondrial dysfunction and increased cellular glucose concentration [42]. As such, these stimuli may in fact represent the underlying causative mechanism behind the atrial remodelling observed in this POAF patient cohort.

### 4.4 Limitations

It is important to consider a number of limitations when interpreting these results. Firstly, the study number remains small and further validation is required in a larger patient cohort. This limited correlation of gene expression with arrhythmia phenotype, although such studies are currently underway. The electrophysiological findings need verification with greater number of sites in the right atrium and importantly the left atrium, which is predominantly responsible for human AF. We chose not to perform LA pacing as the lack of structure function data would have not addressed our primary hypothesis and is under investigation in a separate IRB protocol enrolling mitral valve surgical patients at our centre. Furthermore, POAF represents a specific phenotype of AF in which the LA contribution remains poorly characterised, and we chose patients with no antecedent history or AF and normal LA diameter to exclude undetected atrial substrate. Also, unipolar electrograms were recorded but not analysed due to poor signal:noise ratio. These will not have the direction dependence and electrode orientation effects that bipolar electrodes are subject to. We minimised these factors by using a circular array with electrode pairs in all directions of wavefront propagation.

Furthermore, as this study was not originally designed to examine cellular calcium regulation, although no difference was observed in the expression of the SERCA regulator PLN, these results represent only absolute PLN expression and the phosphorylation state of PLN was not investigated. Phosphorylation of PLN acts to inhibit its binding with SERCA thus removing its inhibitory effect and promoting Ca^2 +^ reuptake into the sarcoplasmic reticulum. As a result, in order to isolate the effect of SLN down-regulation on calcium transport, future work must ensure both PLN and phosphorylated-PLN levels are independently determined. Similarly, a restriction in sample availability did not allow for quantification of myosin heavy chain protein expression and therefore relative changes in MYH6 (and likewise MYH7) expression must now be validated at the protein level [43]. Third, it is important to consider other regulatory mechanisms that may modulate intracellular calcium such as adrenergic stimulation of protein kinase A (PKA) which may not only lead to phosphorylation of phospholamban but also of L-type calcium channels, which in turn increase calcium cycling and speeding up SarcR calcium release [44]. Similarly PKA phosphorylation of Troponin I may also act to lower myofilament sensitivity to Ca^2 +^ thus reducing the contractile response to intracellular calcium load [45].

## 5 Conclusions

Post-operative atrial fibrillation is associated with structural and electrical remodelling present prior to exposure to surgical stress as evidenced by altered paced epicardial electrogram morphology and high frequency spectral components during sinus rhythm, accompanied by changes in the expression of calcium handling proteins. These novel peri-operative upstream substrate changes suggest a cellular mechanism predisposing to post-operative AF, manifest in the electrogram prior to the onset of AF. Further work must now focus on validating these findings in a larger patient cohort, in order to both improve surgical risk prediction and develop the next generation of preventative therapies.

## Funding

This work was supported by the British Heart Foundation (FS/14/46/30907, P26094, P32940), Wellcome Trust (Ref: WT100023MA) and Imperial College Charity (Ref: 5117/R20R).

## Conflicts of interest

None declared.

## Figures and Tables

**Table 1:** Pre-operative demographics: all patients.

	%AF (n = 13)	% nAF (n = 21)	p-Value
Gender (M/F)	9/4	15/6	0.716
Age (yrs)	64.6 ± 11.3	59.6 ± 12.1	0.237
BMI (kg/m^2^)	30.2 ± 6.14	27.5 ± 3.41	0.247
Height (m)	1.62 ± 0.19	1.67 ± 0.08	0.475
Weight (kg)	80.1 ± 3.86	77.7 ± 2.69	0.623
LogEuroSCORE	0.036 ± 0.012	0.019 ± 0.0032	0.188
MI	33%	24%	0.555
Stroke	8.3%	14%	0.614
PVD	17%	4.8%	0.252
PCI	0.0%	0.0%	1.000
LMS disease	25%	33%	0.626
Hypertension	75%	76%	0.939
Hypercholesterolaemia	9.2%	90%	0.909
Family history	45%	65%	0.291
Diabetes	58%	43%	0.392
- *On insulin*	*25%*	*24%*	*0.939*
Smoking	17%	24%	0.629
Alcohol > 10 U per week	25%	25%	1.000

*Pre*-*operative medications*			
Beta-blocker	64%	71%	0.652
Statin	100%	85%	0.177
Diuretic	9.0%	4.8%	0.631
ACE-inhibitors	7.3%	5.2%	0.266
Calcium channel blockers	9.0%	19%	0.461

*Post*-*operative medications*			
Beta-Blocker	70%	87%	0.307
Statin	70%	93%	0.119
Diuretic	100%	100%	1.000
ACE-inhibitors	33%	50%	0.327
Digoxin	10%	0.0%	0.211
Amiodarone*	70%	6.7%	0.001
Proton pump inhibitor	90%	73%	0.307
Magnesium	70%	73%	0.856
Salbutamol (nebulised)	100%	100%	1.000

**Table 2:** Patient demographics: echocardiographic parameters.

Pre-operative echocardiographic parameters					
	n		Mean ± SD		p
AF	Non-AF	AF	Non-AF
LV diastolic diameter (cm)	9	14	4.72 ± 1.06	4.59 ± 0.51	0.7305
LV systolic diameter (cm)	9	13	3.17 ± 0.94	3.17 ± 0.38	0.9964
Fractional shortening (%)	8	11	34.1 ± 10.4	33.9 ± 2.34	0.8998
IVS diastolic thickness (cm)	9	13	1.00 ± 0.18	1.05 ± 0.14	0.5756
LVPW diastolic thickness (cm)	6	12	0.90 ± 0.17	1.02 ± 0.16	0.1911
LA systolic diameter (cm)	9	12	3.83 ± 0.91	3.90 ± 3.54	0.8504
Aortic Root diameter (cm)	8	12	3.19 ± 0.32	3.18 ± 0.60	0.9528
AV peak gradient (mmHg)	9	14	9.72 ± 6.67	7.25 ± 3.26	0.3239
LVOT peak velocity (cm/s)	9	10	89.6 ± 19.4	96.2 ± 21.3	0.4847
LVOT peak gradient (mm Hg)	9	10	4.37 ± 3.56	3.84 ± 1.57	0.6898
MV E′/A′ ratio	8	14	0.83 ± 0.27	0.85 ± 0.24	0.9055
Dilated RA [% (n)]	9	13	22.2% (2)	15.4% (2)	0.683

AF– atrial fibrillation; DM – diabetes mellitus; BMI – body mass index; FH – family history; CHOL – hypercholesterolaemia; HBP – hypertension; PCI – previous percutaneous coronary intervention; PVD – peripheral vascular disease; CVA – stroke; TIA – transient ischaemic attack; MI – myocardial infarction.
